# Sublingual Bacterial Vaccination Reduces Recurrent Infections in Patients With Autoimmune Diseases Under Immunosuppressant Treatment

**DOI:** 10.3389/fimmu.2021.675735

**Published:** 2021-06-04

**Authors:** Silvia Sánchez-Ramón, Lidia Fernández-Paredes, Paula Saz-Leal, Carmen M. Diez-Rivero, Juliana Ochoa-Grullón, Concepción Morado, Pilar Macarrón, Cristina Martínez, Virginia Villaverde, Antonia Rodríguez de la Peña, Laura Conejero, Keyla Hernández-Llano, Gustavo Cordero, Miguel Fernández-Arquero, Benjamin Fernández- Gutierrez, Gloria Candelas

**Affiliations:** ^1^Department of Clinical Immunology, IML and IdSSC, Hospital Clínico San Carlos, Madrid, Spain; ^2^Department of Innovation and Development, Inmunotek S.L., Alcalá de Henares, Spain; ^3^Department of Rheumatology, Hospital Clínico San Carlos, Madrid, Spain; ^4^Department of Rheumatology, Hospital de Móstoles, Madrid, Spain

**Keywords:** mucosal bacterial vaccines, recurrent infections, MV140, MV130, systemic autoimmune disease, biological therapies

## Abstract

**Introduction:**

Conventional or biologic disease-modifying anti-rheumatic drugs (DMARDs) are the mainstay of treatment for systemic autoimmune disease (SAD). Infectious complications are a major concern in their use.

**Objective:**

To evaluate the clinical benefit of sublingual mucosal polybacterial vaccines (MV130 and MV140), used to prevent recurrent respiratory and urinary tract infections, in patients with SAD and secondary recurrent infections following conventional or biologic DMARDs.

**Methods:**

An observational study in SAD patients with recurrent respiratory tract infections (RRTI) and/or recurrent urinary tract infections (RUTI) was carried out. All patients underwent mucosal (sublingual) vaccination with MV130 for RRTI or with MV140 for RUTI daily for 3 months. Clinical evaluation was assessed during 12 months of follow-up after the first dose, i.e., 3 months under treatment and 9 months once discontinued, and compared with the previous year.

**Results:**

Forty-one out of 55 patients completed 1-year follow-up. All patients were on either conventional or biologic DMARDs. A significant decrease in the frequency of RUTI (p<0.001), lower respiratory tract infections (LRTI) (p=0.009) and upper respiratory tract infections (URTI) (p=0.006) at 12-mo with respect to the previous year was observed. Antibiotic prescriptions and unscheduled medical visits decreased significantly (p<0.020) in all groups. Hospitalization rate also declined in patients with RRTI (p=0.019). The clinical benefit demonstrated was concomitant to a significant increase in both anti-*S. pneumoniae* IgA and IgG antibodies following MV130 vaccination.

**Conclusions:**

Sublingual polybacterial vaccines prevent recurrent infections in patients with SAD under treatment with immunosuppressant therapies, supporting a broad non-specific anti-infectious effect in these patients.

## Introduction

Biologic therapies as adjuvants to disease-modifying anti-rheumatic drugs (DMARDs) have revolutionized the treatment of systemic autoimmune disease (SAD). However, increased risk of common and serious infections including bacterial, fungal, and viral infections after biologicals, are a major cause of morbidity and mortality in SAD patients (1–3). The Spanish registry of adverse reactions to biological therapies (BIOBADASER) has found a higher incidence of infections in patients with rheumatoid arthritis (RA) who receive anti-TNF therapies (4). Similar results have been found in a number of different reports (5, 6).

Most common infections affect the upper and lower respiratory tract, skin and the genitourinary tract (1–3). Susceptibility to infection in SAD patients is due to immunological, disease-related and drug-related factors (7). Rheumatic diseases are characterized by immunological alterations, including an impairment of the complement system and a defective response of the innate and adaptive immunity. The increased risk of infection is also linked with the mechanism of action of immunosuppressive therapies. Thus, the use of glucocorticoids (GC) in patients with different autoimmune diseases is associated with an increased risk of infection and hospitalization for pneumonia (6, 7) and local candidiasis (7), as well as increased incidence of opportunistic mycobacterial and viral infections (7). Other immunosuppressive therapies, i.e., TNF inhibitors, may result in initiation or reactivation of granulomatous tuberculosis and fungal infections, as well as increase susceptibility to bacterial infections such as *Pneumococcus* or *Legionella* pulmonary infections, disseminated listeriosis and salmonellosis. Finally, invasive viral infections, mainly herpes virus, are also common (5, 7).

Antibiotics are the mainstay of therapy for infections, but have limitations, such as low penetrance on bacterial biofilms and side effects, including disruption of the microbiota and antimicrobial resistance (8). In addition, antibiotics have no effects on fungal and viral infections. Hence, there is an urgent need of new alternatives or adjuvants for the prophylaxis and treatment of infections (9). This is even more necessary for recurrent or chronic infections in the setting of immunocompromised patients. In this context, recently described trained immunity-based vaccines (TIbV) have been postulated as a promising alternative to reduce recurrent infections (10–12). TIbVs are aimed to elicit not only specific responses to vaccine-related antigens, but to stimulate a broad immune response against unrelated pathogens (10).

MV130 and MV140 are mucosal (sublingual) bacterial vaccines that consist of heat-inactivated whole-cell bacteria. These formulations have shown to confer a non-specific broad-spectrum protection against recurrent respiratory tract infections (RRTI) from bacterial and viral origin (MV130) (11, 13–15) or recurrent urinary tract infections (RUTI) (MV140) (16–21). Both MV130 and MV140 have been described as putative TIbVs (10).

The main objective of this study was to assess the clinical benefit of sublingual polybacterial preparations (MV130 and MV140) in a cohort of SAD patients with recurrent infections using immunosuppressive medication, in routine clinical practice. Besides, the capacity to induce the production of specific antibodies against bacterial antigens contained in the formulations will be also evaluated in these patients.

## Material and Methods

### Patients and Study Design

**Figure 1:**
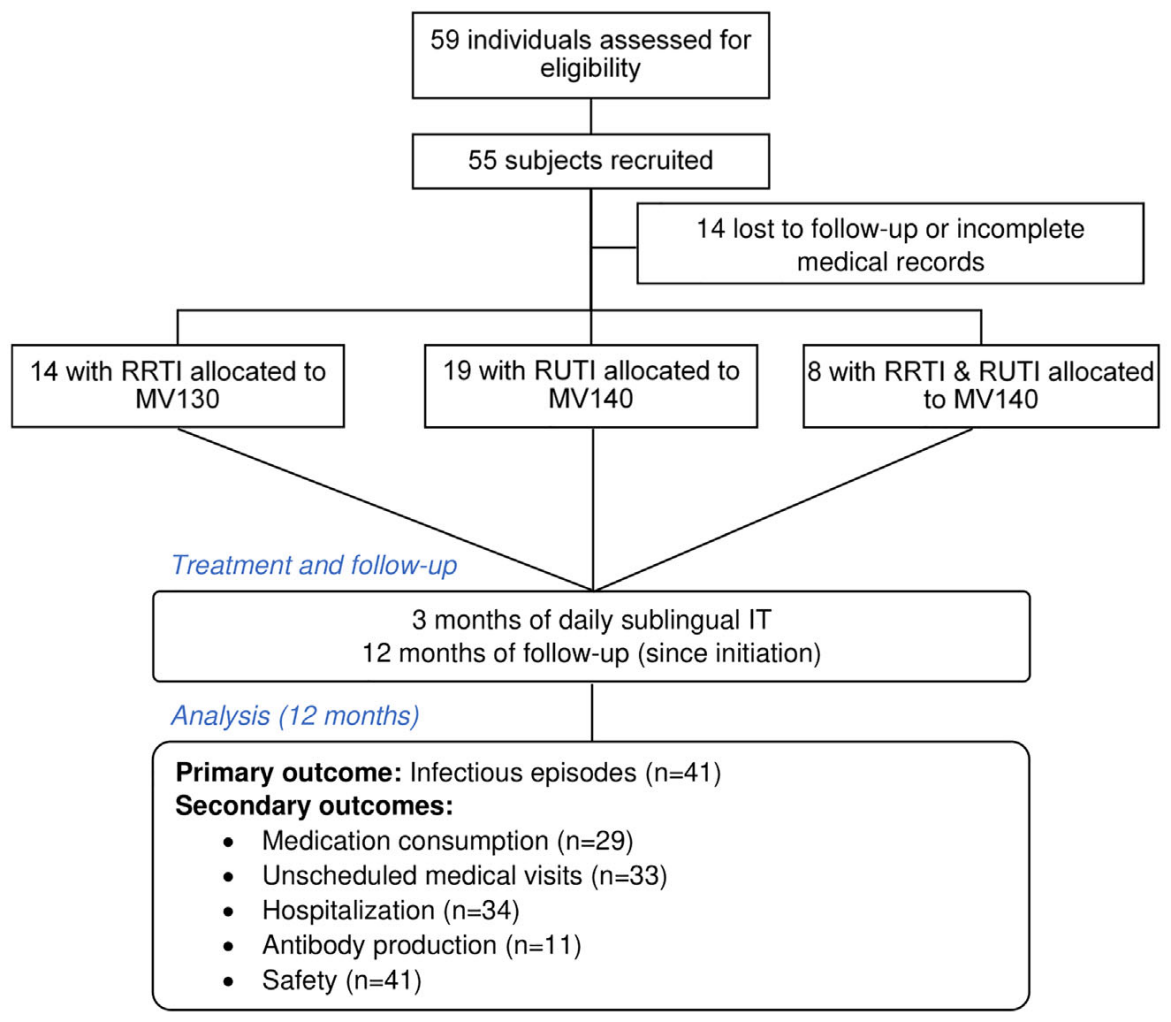
Flow-chart of the study. RRTI, recurrent respiratory tract infections; RUTI, recurrent urinary tract infections; IT, immunotherapy.

A 2-year observational real-life pilot study on a cohort of SAD patients on active therapeutic immunosuppression and recurrent infections was carried out from June 2014 to August 2016. Patients suffering either RRTI (≥3 episodes of upper respiratory tract infection -URTI- or lower respiratory tract infections -LRTI-, at least 1 pneumonia episode/year) or/and RUTI (≥3 UTIs/year) were referred from the outpatient Rheumatology Department to the Clinical Immunology Department, Clínico San Carlos Hospital (Spain) for immunological evaluation. Fifty-five patients were recruited and treated with sublingual vaccination (MV130 or MV140) daily for 3 months. Subjects were allocated in the following groups: SAD patients with RRTI were treated with MV130 and SAD patients with RUTI or suffering from both RUTI and RRTI were treated with MV140 (**Figure 1**).

MV130 (Bactek^®^) and MV140 (Uromune^®^) (both from Inmunotek S.L., Spain) are suspensions of heat-inactivated whole-cell bacteria. MV130 consists of 90% Gram +ve (V101 *Staphylococcus epidermidis*, V102 *S. aureus*, V104 *Streptococcus pneumoniae*) and 10% Gram -ve (V103 *Haemophilus influenzae*, V105 *Moraxella catarrhalis*, V113 *Klebsiella pneumoniae*) bacteria; whereas MV140 contains 25% Gram +ve (V125 *Enterococcus faecalis*) and 75% Gram -ve (V113 *K. pneumoniae*, V121 *Escherichia coli*, and *Proteus vulgaris*). Both MV130 and MV140 contain glycerol, artificial pineapple flavoring, sodium chloride and water for injection, as excipients. MV130 and MV140 are prepared at 300 Formazin Turbidity Units (FTU)/mL (~ 10^9^ bacteria/mL) in spray vials with a metering pump. The delivery route is through the sublingual mucosa and the dose is 2 sprays of 100 μL daily for 3 months, avoiding the concomitant intake of food or beverage. In patients treated with rituximab, bacterial vaccination was started at least 6-months after last dose of rituximab.

The approval for the study was obtained from the Clínico San Carlos Hospital Institutional Research Ethics Committee (16/191-E).

### Outcomes

The rationale for using MV130 and MV140 daily for 3 months was supported by previous publications with both formulations (11, 13, 16–22). We sought to determine the clinical benefit of MV130 in a cohort of patients with SAD and recurrent infections who underwent immunization with MV130 for 3 months. The recommended dose is 2 sprays per day in a single procedure (*i.e.*, one dose is 2 sprays). As part of regular medical follow-up, clinical data regarding infections and immunological data were recorded from each patient the year prior to initiation of vaccination and during the 12 months following the beginning of the treatment with MV130/MV140. The primary outcome of the study was to evaluate the reduction in the number of infectious episodes, as well as safety issues. Secondary outcomes were reduction in the number of antibiotic cycles, unscheduled outpatients’ visits due to infections, and hospitalizations due to infections (**Figure 1**). Unscheduled medical visits were registered by phone interview of each patient and included actual visits to the Emergency Room and urgent calls to the Rheumatology Department for an acute infectious episode. Baseline serum immunoglobulins (Ig) and specific antibody production were performed as per routine clinical practice in our unit. For specific antibody responses, 23-valent pneumococcal vaccine (Pneumo-23), *S. typhi* and diphtheria-tetanus vaccines were administered, and antibody titers were measured after 3-4 weeks, as part of routine evaluation. Serological studies, cultures and imaging techniques were made, as necessary. In a subgroup of patients from which samples were available, specific IgA and IgG anti- *Streptococcus pneumoniae*, anti-*Klebsiella pneumoniae* and anti-MV130 and anti-MV140 antibodies, pre and post MV130 and MV140 treatment were measured. Sera samples were stored frozen at −70°C before further processing.

### Serum IgA and IgG Assays

Sera from selected subjects were obtained before vaccination and at 18-45 months after initiating either MV130 or MV140. Anti-*K. pneumoniae*, anti-*S. pneumoniae*, anti-MV130 or anti-MV140 IgA and IgG were determined by ELISA. Briefly, 96-well non-tissue culture treated plates were pretreated with 100 µL of poly-L-lysine (stock at 0.01%, 1:1000 dilution) (Sigma-Aldrich) for 1 hour under UV light and coated with the appropriate whole-cell heat-inactivated bacteria or polybacterial mixture (300 Nephelometric Turbidity Units -NTU-, ~ 10^9^ bacteria) overnight at 4°C, and subsequently incubated with human sera for 2 hours at room temperature. IgA and IgG antibodies were detected using the following reagents: Biotin rat anti-human IgA or IgG (both from Sigma-Aldrich) and Streptavidin Horseradish Peroxidase (HRP) (Sigma-Aldrich). Peroxidase activity was revealed by addition of o-phenylenediamine dihydrochloride (Sigma-Aldrich) and reaction was stopped with HCl 1N. Plates were read on an ELISA reader at 490 nm (Triturus Elisa, Grifols). Absorbance values (arbitrary units) from each individual subject were normalized to their corresponding pre-vaccination value, thus defined as 1.

### Statistical Analysis

Normal distribution of data was analyzed by means of Shapiro-Wilk test. Continuous variables are expressed as mean ± standard deviation (SD) or median [interquartile range (IQR)], depending on normal distribution, whereas frequency (%) is used for categorical data. For objective parameters of infections’ control, patients served as their own control and paired data were analyzed using the paired t-test for values before and after 1-year. For the clinical variables including infections, antibiotics, unscheduled medical visits and hospitalizations, analysis was carried out with Wilcoxon signed-rank test, since these variables did not follow a normal distribution and comparison was conducted between two sets of scores that came from the same participant. Statistical significance for post-vaccination data (IgA and IgG responses) was calculated using one sample t-test with 1 as theoretical mean value. SPSS V 15 and GraphPad Prism software (GraphPad Software, La Jolla, CA, USA version 8) were used. A two-sided p-value <0.05 was considered statistically significant. Evaluable population for effectiveness included any recruited subjects who completed the whole study period (treatment and follow-up) with full information on the primary outcome. Evaluable safety population included enrolled subjects who initiated the assigned treatment. No additional factors were considered in the analysis.

## Results

### Epidemiologic and Baseline Data

Between June 2014 and August 2016, a total of 59 SAD patients with RRTI, RUTI or both RRTI and RUTI referred from the outpatient Rheumatology Department to the Clinical Immunology Unit were evaluated. Fifty-five of them were included in the study and 41 of them completed 1-year follow-up after sublingual vaccination with MV130 or MV140 (**Figure 1**). The mean age (± SD) of patients was 54.68 ± 14.66 years and 38 out of 41 (92.7%) were females. According to the patient’s diagnosis, their SAD was classified as: RA (n=18; 43.9%), SLE (n=8; 19.5%), mixed connective tissue disease (MCTD) (n=3; 7.3%), and miscellanea (n=12; 29.3%) (**Table 1**). Only 3 out of 55 (5.4%) patients were current smokers and 8 (14.5%) were former smokers. None of the patients were on antibiotic prophylaxis. Of the 55 SAD patients, 78% reported at least one comorbidity. The prevalence of specific comorbidities was: hypercholesterolemia (13; 23.6%); hypertension (9; 16.3%); chronic obstructive pulmonary disease (7; 12.7%); diabetes (3; 5.4%).

**Table 1:** Demographic and clinical characteristics of subjects at baseline (n=41).

	Mean ± SD or N (%)
**Demographic characteristics**	
Age (years)	54.68 ± 14.66
Age range	21 – 81
Sex (M/F)	3/38 (3.7%/92.7%)
**Rheumatic diseases**	
Rheumatoid arthritis	18/41 (43.9%)
Systemic lupus erythematosus	8/41 (19.5%)
Mixed connective tissue disease	3/41 (7.3%)
Others (miscellaneous)	12/41 (29.3%)
**Recurrent infectious diseases**	
Recurrent respiratory tract infections	14/41 (34.1%)
Recurrent urinary tract infections	19/41 (46.3%)
Both	8/41 (19.5%)
**Immunological status**	
Antibody deficiency	8/41 (19.5%)
Hypogammaglobulinemia	3/41 (7.3%)
Others (miscellaneous)	5/41 (12.2%)

Data is expressed as mean ± SD or frequency (%) of total subjects. M, male; F, female.

All patients were on DMARDs at baseline, being methotrexate the most frequent. Twenty-eight (48.72%) needed biologicals associated to conventional DMARDs, being anti-TNF-α monoclonal antibodies the most frequently used (38.0%), followed by anti-CD20 (14%) and tocilizumab (6.8%). Additionally, 87.2% patients were on low dose steroids (90% on 2.5mg daily, 10% on 5mg daily). The most common recurrent infections observed were RUTIs (46.3%), followed by RRTI (34.1%); 19.5% of the subjects were affected of both RUTI and RRTI (**Table 1**). Concerning immunological status, eight patients (19.5%) presented antibody deficiency and 3 subjects hypogammaglobulinemia (7.3%) (**Table 1**).

At baseline, 3 out of 41 SAD patients (7.3%) presented with low IgG (mean 4.55 g/L); 2 (4.8%) patients low IgA (mean 0.04 g/L) and 3 patients (7.3%) low IgM (mean 0.055 g/L). With respect to specific antibody responses production after pneumococcal polysaccharide (PCP) vaccination (Pneumo-23), 22 out of 41 (53.6%) patients showed low antibody anti-PCP responses; 23 out of 41 (56.0%) patients showed low antibody responses to tetanus toxoid vaccination; and 26 patients out of 41 patients (63.4%) showed low anti-S. *typhi* antibody production after *S. typhi* vaccination.

### Clinical Endpoints Following Sublingual Vaccination

**Figure 2:**
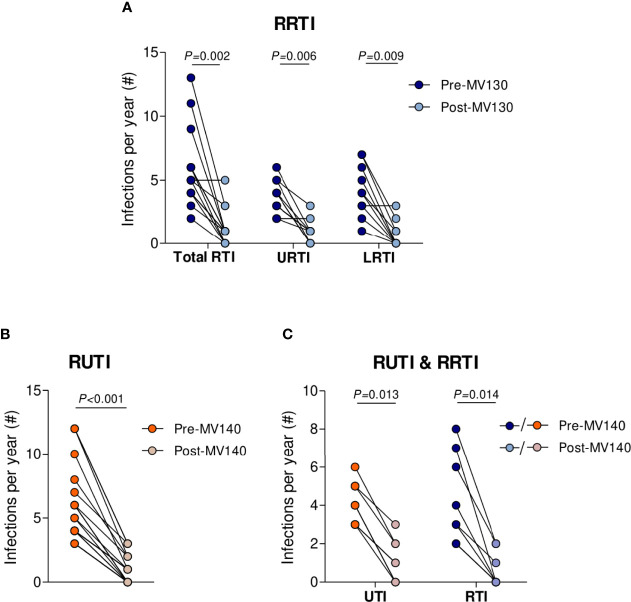
MV140 and MV130 induce a significant fall-off in the incidence of recurrent respiratory and urinary infections. **(A-C)** Number of infectious episodes scored 1 year prior to vaccination and throughout 12 months after the initiation of immunotherapy (MV130 or MV140) in subjects suffering RRTI **(A)**, RUTI **(B)** or both **(C)**. RRTI: Recurrent respiratory tract infections, either upper (URTI), lower (LRTI) or both (total RTI); RUTI: Recurrent urinary tract infections. Data from 14 **(A)**, 19 **(B)** or 8 **(C)** subjects are shown. Lines link paired values. Normal distribution was evaluated using the Shapiro-Wilk test. *P* values were calculated using Wilcoxon signed-rank test.

The frequency of infections was recorded for each patient in the year prior to, and 12 months after initiating MV130/MV140 treatment. Globally, a statistically significant decrease in the frequency of RRTI [5.00 (4.00-6.75) to 1.00 (0.00-1.50), p=0.002] was observed following MV130 administration (**Figure 2A**). Further analysis demonstrated that this reduction was also noted when RRTI were split into URTI [4.00 (3.00-5.00) to 1.00 (0.00-1.00), p=0.006] and LRTI [4.00 (2.75-6.25) to 0.00 (0.00-1.25), p=0.009] (**Figure 2A**). For URTIs, 10 out of 11 (90.9%) cases reduced above 40% the number of infections. Nevertheless, just 1 out of 11 patients evaluated affected with sarcoidosis did not show a beneficial response in reducing the number of infections. As for LRTI, 6 out of 10 (60.0%) patients had a total reduction, 3 subjects a decrease above 70%, with only one patient without improvement on the infectious rate (**Figure 2A**).

As for RUTI, a significant decline in the infectious rate was observed following treatment with MV140 with respect to the previous year [5.00 (4.00-8.00) to 1.00 (0.00-2.00), p<0.001] (**Figure 2B**). In these lines, 8 out of 19 cases (42.1%) showed a total reduction and 11 out of 19 (57.9%) reduced the number of UTI over 50%. Eight SAD patients that suffered from both RRTI and RUTI were put on MV140 with the same treatment schedule, evidencing a significant drop in RRTI and RUTI following MV140 treatment (p=0.013 and p=0.014, respectively) (**Figure 2C****)**.

**Figure 3:**
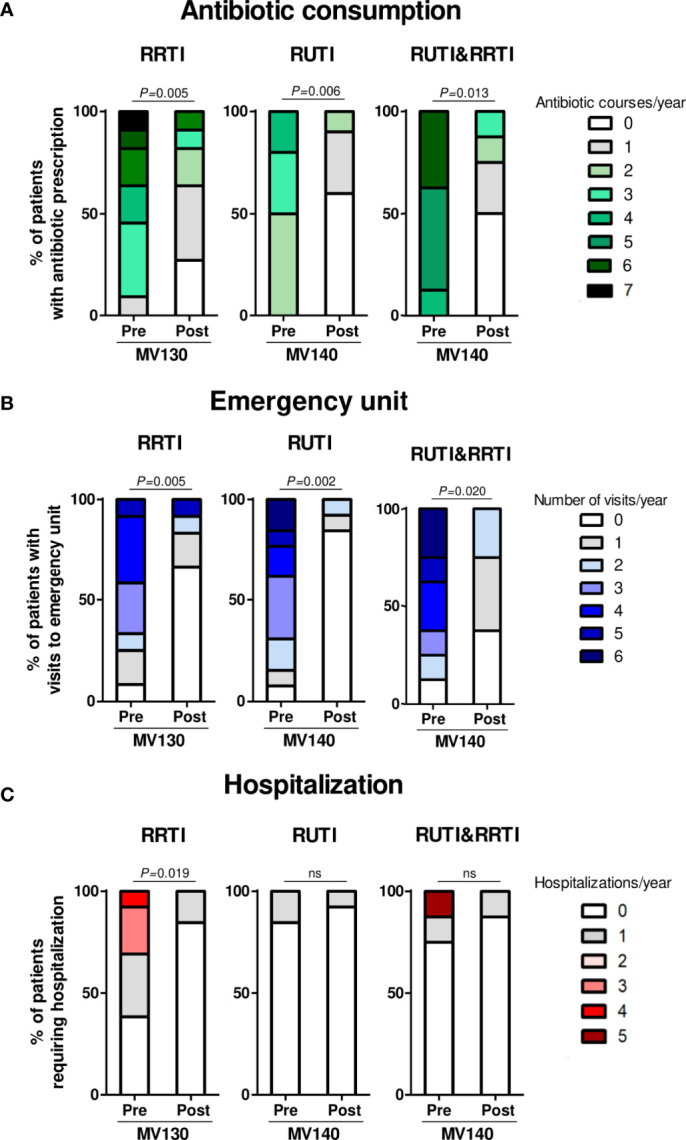
Prophylaxis with MV130 and MV140 reduce the consumption of healthcare resources. **(A–C)** Antibiotic consumption **(A)**, unscheduled medical visits (emergency unit and specialist) **(B)** and hospitalization **(C)** in subjects suffering RRTI (left panel), RUTI (middle panel) or both (right panel), during the year prior (pre) and after (post) the initiation of the treatment (MV130 or MV140). RRTI: Recurrent respiratory tract infections; RUTI: Recurrent urinary tract infections. Bars show the relative abundance of the number of antibiotic courses **(A)**, unscheduled medical visits **(B)** or hospital admissions **(C)** in the total of subjects recorded. Data from 29 **(A)**, 33 **(B)** or 34 **(C)** subjects, receiving either MV130 or MV140 according to their pathology are shown. Normal distribution was evaluated using the Shapiro-Wilk test. *P* values were calculated using Wilcoxon signed-rank test, ns, non-significant.

The median number of antibiotic cycles’ prescriptions significantly decreased in any pathology following vaccination, from 4.00 [3.00-5.00] to 1.00 [0.00-2.00] (p=0.005) in RRTI patients, from 3.50 [3.00-4.25] to 0.00 [0.00-1.00] (p=0.006) in RUTI individuals, and from 5.00 [5.00-7.00] to 0.50 [0.00-1.75] (p=0.013) in patients suffering both types of infections. **Figure 3A** shows the relative frequency of prescriptions according to infection site, respiratory or urinary tract. Overall, from 29 patients where antibiotic information was reliable, 13 (44.8%) individuals showed a 100% reduction in antibiotic consumption.

The median unscheduled medical visits (Emergency Unit or specialist) due to any recurrent infection were significantly reduced, from 3.00 [1.25-4.00] to 0.00 [0.00-1.00] (p=0.002) in RRTI patients, from 3.00 [2.00-4.50] to 0.00 [0.00-0.00] (p=0.005) in RUTI individuals, and from 4.00 [2.25- 5.75] to 1.00 [0.00-1.75] (p=0.020) in patients suffering both RRTI and RUTI. Globally, 22 out of 33 patients (66.7%) showed no need of unscheduled visits to the healthcare system following bacterial immunotherapy. **Figure 3B** shows the relative frequency of unscheduled medical visits in patients with RRTI, RUTI or both.

Finally, the median hospitalization’s rate significantly decreased from 1.00 [0.00-3.0] to 0.00 [0.00-0.00] (p=0.019) in patients with RRTI, who are more at risk of infectious complications and thus, more susceptible to hospitalization. Herein, the number of hospital admissions was reduced in all individuals requiring hospitalization in the previous year. Additionally, three patients with antibody production deficit and pneumonia required intravenous Ig. **Figure 3C** shows hospitalization rate according to tissue infection target (airways or urinary tract).

Regarding safety, no adverse effects or SAD relapses were noted during the 1-year follow-up period since the initiation with MV130 or MV140 immunotherapy. Thus, neither local nor systemic adverse reactions related to MV130/MV140 were reported.

### Specific Humoral Responses to MV130 and MV140

**Figure 4:**
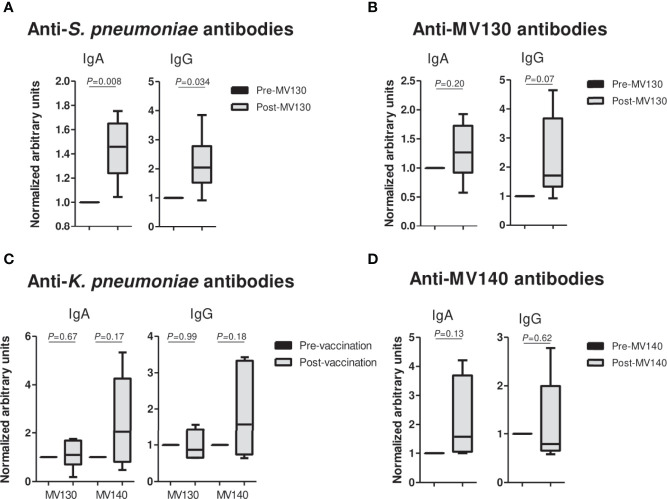
Prophylaxis with mucosal bacterial vaccines increases serum antibody production. **(A–D)** Serum IgA (left panels) and IgG (right panels) antibodies against the specified pathogens **(A, C)** or the bacterial mixture **(B, D)**, collected from subjects before vaccination and at 18-45 months after initiating either MV130 or MV140 immunotherapy. Data in each individual subject are normalized to the corresponding pre-vaccination value. Results from N=6 (MV130) or N=5 (MV140) individuals are shown as mean+SEM for each immunoglobulin. *P* values were calculated using one sample *t*-test with 1 as theoretical mean value.

Finally, to assess in these patients whether MV140 and/or MV130 induce specific systemic humoral responses against bacteria included in their formula, blood samples from a small number of subjects (n= 6 for MV130, and n=5 for MV140) were assayed for specific IgG and IgA. A significant increase in both anti-*S. pneumoniae* IgA and IgG antibodies following MV130 immunization was observed. *S. pneumoniae* accounts for 60% of bacteria included in MV130 and *K. pneumoniae* is included in both MV130 and MV140 (**Figure 4**). A similar tendency was observed when anti-MV130, anti-MV140 and anti-*K. pneumoniae* serum antibodies were tested.

## Discussion

Morbidity and mortality in patients with SAD are associated to infectious complications that are in part due to intrinsic disease-related immune dysregulation but also associated with immunosuppressive treatment, including biologic therapies (5, 7). Therefore, prophylactic strategies that prevent or ameliorate serious infectious risk are a priority for these patients.

Our proof-of-concept real-life study shows that mucosal (sublingual) vaccination with MV130 or MV140 is safe and significantly reduced the infection rate, antibiotic consumption and use of healthcare resources in actively immunosuppressed SAD patients with recurrent infections. To our knowledge, this is the first study of using mucosal vaccines in SAD patients to prevent recurrent infections. Previous studies have demonstrated a decrease in infectious episodes of different etiology, including bacteria and viruses, in immunocompetent patients with RRTIs following MV130 administration (13, 14). Furthermore, our results are further supported by recently published data in common variable immunodeficiency subjects suffering from RRTIs, where MV130 treatment significantly reduced the number of episodes of respiratory infection, antibiotic uptake, and unscheduled medical visits (11). On the other hand, different retrospective and prospective studies indicate that MV140 protect women suffering from uncomplicated RUTI, increasing the probability of UTI-free status and/or reducing the frequency of UTIs (16–21).

Currently, there are not conventional vaccines available for certain pathogens that cause recurrent infections, such as those responsible for many respiratory and urinary tract infections. The efficacy of MV130 conferring protection against unrelated pathogens was pointed out in a clinical trial with children suffering from recurrent wheezing, a disease mainly triggered by viral infections. In this clinical trial, MV130 group showed a significant reduction in the number and duration of wheezing attacks compared to placebo (15). In this line, in a former pilot study in a cohort of patients with RRTIs, one of the subjects had 12 episodes of labial and nasal herpes virus prior to MV130 administration, this number dropped to 3 episodes following MV130 treatment (14). Similarly, there is evidence that MV140 does not only protect against urinary infections driven by pathogens contained in its vaccine formulation, but also against unrelated bacteria (19). In our cohort, a relevant clinical finding was that patients with both recurrent urinary and respiratory tract infection reduced both kind of infectious episodes when they were treated with MV140, reinforcing a broad-spectrum, non-specific immunological effect for these preparations. Thus, the beneficial effects of these vaccines might solve a clinical problem faced by rheumatologists treating SAD patients with immunosuppressive treatment, further adding to previous studies on non-immunosuppressed patients.

MV130 and MV140 contain whole-cell inactivated bacteria. Gram positive and Gram negative bacteria act in a synergistic and complementary way in the activation of innate immunity (23). The new concept of innate immune memory, also called trained immunity, is defined as a functional reprogramming of innate immune cells by certain pathogens or their components, including specific vaccines, leading to heterologous protection against infection (24, 25). This process provides the potential to identify novel prophylactic targets that protect from secondary infections (26), and to assay novel approaches in vaccinology to develop TIbVs (10). The non-specific effect of MV130 and MV140 in clinical studies conferring protection against a broad range of pathogens has led to their inclusion as putative TIbVs (10). The mechanism of action immunomodulating the immune response of both mucosal bacterial preparations has been further studied *in vivo* and *in vitro* (23, 27, 28). In this regard, when MV130 and MV140 are administered sublingually to mice, a potent systemic Th1/Th17 and IL-10 response is observed (23, 27). In the case of MV130, this immune response has been also observed against unrelated stimuli (27). In addition, a role for both bacterial formulations triggering trained immunity has been also assessed (Brandi et al., submitted) (29). A possible detrimental role for trained immunity triggering or enhancing autoimmune and autoinflammatory diseases has previously been speculated (30); however, this was not the case in our study. None of the subjects included in the study had any autoimmunity outbreak following mucosal immunotherapy. We believe the high levels of IL-10 released by innate and adaptive immune cells *in vitro* and *in vivo* following MV130 and MV140 may account, at least partially, for this protection (23, 27, 28).

Besides clinical benefit, a significant increase in both anti-*S. pneumoniae* IgA and IgG antibodies was observed following MV130 immunization. *S. pneumoniae* accounts for 60% of bacteria included in MV130, pointing to an induction of humoral specific response upon bacterial immunotherapy. A similar tendency was observed when anti-MV130, anti-MV140 and anti-*K. pneumoniae* serum antibodies were tested. This finding is particularly important given the active immunosuppression in these patients and that about half of them were above 65 years-old (31). A rapid decrease in antibody levels after the vaccination has been reported in groups of elderly or immunocompromised patients (32, 33). We speculate that the increase in IgA levels may be explained due to the mucosal delivery route of these vaccines. Mucosal immunization has been proven to be more effective than other routes at eliciting a strong mucosal and systemic immune response (34). In these lines, mucosal delivery of bacterial lysates provided protection against respiratory viral infections enhancing mucosal and systemic antibody production in mice (35). Of note, these results support previous findings that indicated that patients with primary immunodeficiency significantly increase their titles of anti-pneumococcus IgA antibodies following MV130 treatment (11).

Finally, although the design of our work did not address economic or quality of life as previous reports have assessed (11), we anticipate an improvement on patients’ quality of life, reduced antibiotic resistance, and health care saving concomitant to the clinical benefit herein demonstrated.

In conclusion, these data support the hypothesis that MV130/MV140 immunization is safe and confers clinical benefit against a broad-spectrum of infections of diverse etiology; the clinical improvement is concomitant to an enhanced immune response illustrated by an increased in specific antibody production. Validation of these results in a prospectively designed clinical trial with a larger sample size are warranted to better understand the clinical relevance of these findings.

## Data Availability Statement

The raw data supporting the conclusions of this article will be made available by the authors, without undue reservation.

## Ethics Statement

The studies involving human participants were reviewed and approved by the Institutional Research Ethics Committee from Hospital Clínico San Carlos. Written informed consent for participation was not required for this study in accordance with the national legislation and the institutional requirements, since data were collected conforming medical records as per routine clinical practice.

## Author Contributions

Study conception and design: SS-R and GCa. Patient recruitment and data collection: SS-R, GCa, JO-G, LF, CMo, PM, CMa, VV, AR-P, KH-L, GCo, MF-A, and BF-G. Analysis and interpretation of data: SS-R, GCa, JO-G and CD-R. Drafting the manuscript: SS-R, GCa, JO-G, PS-L, CD-R and LC. All authors attest they meet the ICMJE criteria for authorship. All authors contributed to the article and approved the submitted version.

## Conflict of Interest

PS-L, CD-R and LC are employees of Inmunotek S.L. SS-R has received in the past a fee as speaker from Inmunotek S.L.

The remaining authors declare that the research was conducted in the absence of any commercial or financial relationships that could be construed as a potential conflict of interest.
