# Supplementary material for: Sublingual Bacterial Vaccination Reduces Recurrent Infections in Patients With Autoimmune Diseases Under Immunosuppressant Treatment
Source: Front Immunol. 2021 Jun 4;12:675735. doi: 10.3389/fimmu.2021.675735 (PMC8212043; doi:10.3389/fimmu.2021.675735)
Supplement: Supplementary file 1 [file Table_1.docx]

**Supplementary Material**

**Table S1**. Demographic and clinical characteristics of antibiotic-requiring subjects at baseline (n=29).

| **Baseline characteristics** |  |
| --- | --- |
| **Demographics** |  |
| Age (years, mean ± SD) | 56.30 ± 14.48 |
| Age range | 24-81 |
| Sex (M/F) | 2/27 (6.9%/93.1%) |
| **Rheumatic diseases, n (%)** |  |
| Rheumatoid arthritis | 13/29 (44.8%) |
| Systemic lupus erythematosus | 5/29 (17.2%) |
| Mixed connective tissue disease | 2/29 (6.9%) |
| Others (miscellaneous) | 9/29 (31.0%) |
| **Recurrent infectious diseases, n (%)** |  |
| Recurrent respiratory tract infections | 11/29 (37.9%) |
| Recurrent urinary tract infections | 16/29 (55.2%) |
| Both | 2/29 (6.9%) |
| **Immunological status, n (%)** |  |
| Antibody deficiency | 4/29 (13.8%) |
| Hypogammaglobulinemia | 2/29 (6.9%) |
| Others (miscellaneous) | 4/29 (13.8%) |

Data is expressed as mean ± SD or frequency (%) of total subjects. M, male; F, female.
